# Young Adults' Needs in Social Robot Assisted Medication Counselling: Applying Peplau's Interpersonal Relations Model

**DOI:** 10.1111/nup.70074

**Published:** 2026-03-01

**Authors:** Malin Andtfolk, Sara Rosenberg, Susanne Hägglund, Mattias Wingren, Andreas Lundell, Linda Estman

**Affiliations:** ^1^ Department of Health Sciences, Faculty of Science and Engineering Åbo Akademi University Vaasa Finland; ^2^ Experience Lab, Faculty of Education and Welfare studies Åbo Akademi University Vaasa Finland; ^3^ Department of Information Technology, Faculty of Science and Engineering Åbo Akademi University Vaasa Finland; ^4^ Department of Caring and Ethics, The Faculty of Health Sciences University of Stavanger Stavanger Norway

**Keywords:** interpersonal relation, medication counselling, needs, peplau, social robot, young adult

## Abstract

The integration of robots into healthcare is an advancing field, with potential to enhance patient care, for example in medication counselling. Peplau's Interpersonal Relationship Model was adapted as a framework for the aim to explore the needs of young adults in relation to social robots in medication counselling. Peplau's model outlines the phases of the nurse‐patient relationship and is emphasised through three key phases, such as Orientation phase, Working phase and Termination phase. Qualitative interviews with six young adults were analysed using deductive reflexive thematic analysis. The results suggest the following: In the orientation phase, the robot should offer reliable and clear communication and a neutral appearance to the young adult to create a trusting relation. In the working phase, the robot should utilise the individuals’ resources to address their medication‐related needs and begin to anticipate goals beyond the immediate problem. Finally, in the termination phase, the robot facilitates the transition to independence for the young adult to manage own medication without relying on the robot. This research contributes to the broader discourse on the intersection of technology and human caring, emphasising the importance of maintaining interpersonal relation in the digital age. Insights from this study informs the design and implementation of social robots in healthcare, ensuring that they effectively meet the needs of young adults and complement medication counselling.

## 1 Background

The global social and healthcare sector is currently facing both immediate and long‐term challenges. These include a shortage of care professionals such as pharmacists (Pharmaceutical Group of the European Union 2024; European Parliament 2025), and escalating healthcare costs (Roberts 2009; Lorenzoni et al. 2019). As the healthcare landscape evolves, the importance of adapting to new technologies becomes increasingly evident. Social‐ and healthcare, including pharmacies, have undergone extensive digitalisation in recent years (Pharmaceutical Group of European Union 2019). This transformation has brought about socio‐technical challenges (Angelis and Braccini 2019), and the implementation of new digital working methods requires new ways of interactions and access to medications (Suomen Apteekkariliitto 2022). A promising new way of interaction in health and social care is social robotics, where social robots are developed to support human interaction through artificial intelligence. A social robot is designed to interact, perform services and operate in a natural, efficient and a socially acceptable way with humans in humanlike environments (Breazeal et al. 2016). However, for social robots to meet safety requirements, studies are needed on what contributes to increased trust among staff and clients (Suha and Sanam 2023).

Ensuring medication safety during counselling is a critical aspect of healthcare. According to the Ministry of Social Affairs and Health (2022) in Finland, safe medication treatment can be compromised at any stage of the medication treatment process and in any unit that carries out medication treatment or dispensing, i.e. also in pharmacies. Compromising the safety of medication treatment can cause serious side effects, increase costs (Assiri et al. 2019) and can cause confusion or poor health outcomes for patients (Gilson et al. 2019). Researchers believe that it is possible to prevent most incidents that occur in connection with medication treatment (Härkänen 2014; Härkänen et al. 2020). Medication information is available, but the information is often shared across different healthcare providers without proper coordination, which can cause problems for clients (Fimea 2012). Lack of time, inadequate knowledge (e.g., language skills) or costs can be obstacles to using information sources. Although promoting the safety of medication treatment, such as counselling, is included in the public service promise in Finland (Ministry of Social Affairs and Health 2022), medication safety remains a major challenge nationally (Mäkinen et al. 2025).

Previous research shows that trust in social robots affects both collaboration between humans and robots and the sustainability of their use (Hägglund et al. 2024) and that trust robots with artificial intelligence influenced by, among other things, the model's ability to act multilingually and multiculturally (Joshi et al. 2023). In addition, there are indications that trust is a prerequisite for at least social sustainability (Hägglund et al. 2024). One of the most debated areas for social robot technology is its impact on social inclusion and access to services. If social robots are to be used in healthcare, they need to be adapted to the local cultural and linguistic context (Hägglund et al. 2023). There are many ways in which humans and robots can communicate. These include visual tools like graphical user interfaces or interaction points in augmented reality, as well as nonverbal cues like facial expressions, hand gestures, speech, natural language, physical interaction, and touch (haptics). The overarching aim of Human‐Robot Interaction is to make this interaction feel as natural as possible (Arif et al. 2017). As there are still knowledge gaps on how a social robot can be designed in a sustainable and safe manner within the strictly regulated pharmacy environment, holistic research is needed that broadens the view of relations towards robots implemented in social and healthcare contexts (Zafrani and Nimrod 2019). Thus, it is important to study and ensure that social robots for medication counselling meet individual needs and safety requirements, provide accurate and verified information, protect user data, and are transparent in their functions. In addition, some women have reported discomfort when purchasing certain medications than others, such as emergency contraceptive pills (ECPs), at pharmacies. Turnbull et al. (2021) saw that pharmacy staff were often perceived as judgmental and unsupportive, and that some women preferred travelling to another city to avoid encountering acquaintances at the pharmacy, and others waited until the pharmacy was empty to inquire about ECPs.

Recommendations for European pharmacies by 2030 emphasise ensuring safe, quick, and equitable access to medications through well‐trained staff governed by regulations, practices, and ethics (Pharmaceutical Group of European Union 2019). However, there is a need to explore new dimensions of technological support structures, such as social robots, which can help alleviate the workload of healthcare personnel without compromising ethics, safety, and sustainability for the individuals. In previous research on social robots, the focus has often been on interactions between humans and robots. However, within the field of caring and nursing science, it is essential to understand the significance of relationships, especially in contexts where care, trust, and communication about health and medications are central. Therefore, the aim of this research was to explore the needs of young adults in relation to social robots in medication counselling at pharmacies using Peplau's Interpersonal Relationship Model. Peplau's model emphasises the importance of building trustful relationships between caregivers and patients. By applying this model, the research can better understand how social robots can support and enhance these relationships, thereby meeting the specific needs of young adults regarding medication counselling.

## 2 Theoretical Background

This study applies Peplau's theory as a framework to understand young adults' needs—from the initiation of the relationship with the robot, through the counselling process, to its conclusion. Peplau (1952) middle range theory was seen as suitable for studying human‐robot interaction, as it emphasises the development of trust, communication and information as well as patient autonomy. This highlights how robots can support and respect the individual's health goals and autonomy, which aligns with the aims of this research. Peplau wanted nurses to use situations in nursing as a source to understand concepts unique of nursing (Peden 2001) and initially referred to an early version of the theory as ‘talking to patients’. However, this idea of talking to the patients evolved into the therapeutic interpersonal relationship theory, a framework suitable to all individuals under care. Peden (2001) noted that ‘Peplau's work introduced a woman ahead of her time, and today her theory aligns with modern nursing influences that have heightened nurses' awareness of the knowledge‐rich context of practice, particularly at the patient level’ (pp. 62‐63).

According to Peplau (1952), nursing is defined as the therapeutic relationship between two individuals, emphasising that the nurse must engage with the patient intentionally. She stressed that the terms ‘relations’ and ‘relationships’ should not be used interchangeably as differences between these lie in the depth and development over time. She suggests that the dynamics between two individuals can be quite complex and difficult to define. Peplau viewed communication as a tool to encourage positive changes in patient behaviours. Her model defined health in a broad sense as the progressive development of personality and human processes. In each phase of the nurse‐patient relationship, both the nurse and the patient engage in purposeful behaviours that reflect the evolving nature of their interaction. The nurse utilises the relationship to assess the patient's psychological, emotional, and spiritual needs, drawing on communication skills, personal strengths, and a deep understanding of human behaviour. The goal is to achieve positive outcomes that benefit the patient. Trust is established in any patient care setting when the patient feels assured of the nurse's integrity and reliability. Through the nurse‐patient relationship, the nurse is also able to demonstrate empathy, which is the ability to understand and perceive the patient's emotions and meaning and effectively communicate that understanding back to the patient (Fawcett 2010).

According to Peplau (1952), the nurse‐patient relationship can be expressed as a process with three overlapping phases: (a) the orientation phase, (b) the working phase, and (c) the termination phase. It is crucial for the nurse to establish clear boundaries and maintain them throughout all phases of the process. During *the orientation phase*, the patient exhibits health‐seeking behaviours, and the nurse is recognised as someone capable of helping the patient. This phase builds a trusting relationship, as the nurse gathers information, conducts initial assessments of the patient's needs, potential, and interests, and evaluates the patient's susceptibility to fear or anxiety (Fawcett 2010). Next is the *working phase*, where most of the relationship's work occurs. This phase includes two sub‐phases: *identification and exploitation*. In the identification stage, the nurse may assume roles such as care provider, educator, or counsellor (Fawcett 2010). The nurse uses professional skills to address health issues, while the patient recognises the nurse's consistent support and empathy. As the patient becomes more independent in care, power shifts from the nurse to the patient. During this exploitation sub‐phase, the nurse also begins planning for discharge, focusing on education and leadership (Peplau 1952). During the *termination phase*, the nurse and patient end their relationship. During this phase, the nurse reviews the discharge plan and helps the patient prepare for new, socially interdependent relationships. Peplau believed that this phase promotes the patient's ability to become more self‐reliant and lead a healthier, more productive life (Fawcett 2010).

## 3 Methods

### 3.1 Study Design and Setting

The article draws on data from the multi‐disciplinary PharmAInteraction project exploring whether socially assistive robots at pharmacies may strengthen patient and medication safety (cf. Andtfolk et al. 2022; Andtfolk et al. 2024; Rosenberg et al. 2024; Hägglund et al. 2024; Wingren et al. 2025). Data is gathered from the first stages of a design process, where the project developed and tested a prototype robot application with potential end participants and pharmacists. In Finland, ECPs are over‐the‐counter medications, but they require extra medication counselling. In this study, the social robot Furhat acted without the immediate presence of a pharmacist (Fimea n.d).

Due to strict regulations on medication counselling, a controlled system based on rules was designed. The dialogue structure was developed in collaboration with pharmacists using methods such as role play and Hierarchical Task Analysis (Dreger et al. 2023). Based on the responses to these variables, the Furhat robot provided tailored medication options and delivered information about each option's mechanism, instructions for use, and potential side effects. Finally, participants were invited to select from several available alternatives. In accordance with Finnish legislation (Finlex 1987), the robot also communicated the availability of interchangeable products and their respective prices, as this information is legally required to be shared with customers (cf. Hägglund et al. 2024).

Qualitative interviews with a deductive approach were considered suitable based on the study purpose, allowing for a structured but nuanced understanding of how the theory applies in care practice. According to Braun and Clarke (2022), the deductive research approach offers a means to shape the data analysis by an existing theoretical construct. Peplau's theory applied here serves as a lens for examining the data.

### 3.2 Participants and Data Collection

Purposeful sampling (Palinkas et al. 2015) was used to recruit participants who were Swedish‐speaking Finns, aged 20–40, female, and somewhat experienced with technology. Prior knowledge of ECPs was not a requirement. Six female participants, aged 29–37 (mean 30.5), participated in the study. None had previously interacted with Furhat. The study participants interacted with Furhat individually in a simulated pharmacy setting, following consent forms and privacy notices. A researcher was available in case of technical issues, and the human‐robot interaction was video recorded for future analysis. This article focuses on the qualitative interview data from the experience with Furhat, providing the medication counselling. Immediately after the interaction, qualitative data were gathered through six semi‐structured post‐test interviews, each lasting between 24 and 46 min, and recorded for analysis.

### 3.3 Analysis

Data were analysed using deductive, reflexive thematic analysis (Braun and Clarke 2022), focusing on broad thematic patterns across all data from a constructivist perspective. Reflexive thematic analysis is particularly suited for studies like this due to its flexibility and accessibility (Braun and Clarke 2022, p. 261). The deductive analysis was carried out manually, concentrating on exploring the realities and meanings of the needs in relation to social robots raised by the participants. The analysis process began by reviewing the interviews multiple times and an initial analysis of the interview transcripts by code categorisation was conducted by three of the authors (M.A., S.H., L.E.). The same authors collaborated to enhance the validity and reliability. Initial themes put forward by the first author (M.A.) were finalised and confirmed as themes at the end of the analysis process through discussions, including all the researchers in the team (M.A., S.R., S.H., M.W., A.L., L.E.). The themes developed from the data content were shaped by the theoretical framework provided by Peplau (1952).

After reviewing the codes and dataset, the authors identified three main themes related to participants' needs in relation to social robots. Each theme is expressed through Peplau's interpersonal relation theory, symbolising the dynamic and evolving nature of human‐robot relations. Illustrative participant quotes help highlight the driving motives behind their willingness to build a relationship with the robot in medication counselling.

## 4 Results

**Figure 1:**
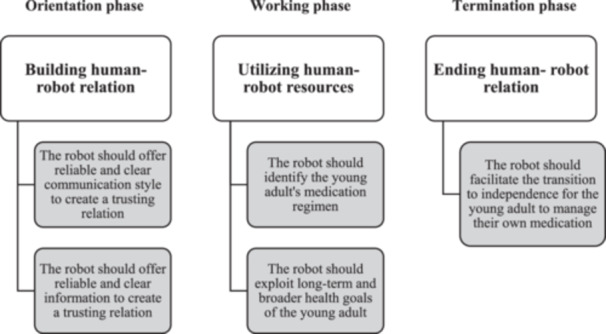
A thematic map summarising young adults' needs in relation to social robots in medication counselling.

The result of this study focuses on exploring the needs of young adults in relation to social robots in medication counselling using Peplau's Interpersonal Relationship Model. The study findings are organised in a thematic map (Figure 1) based on the theoretical framework used in the data analysis, highlighting the main themes *Building human‐robot relation, Utilising human resources*, and *Terminating human‐robot relation*. In addition, five subthemes was found such as *Offer reliable and clear communication style, Offer reliable and clear information, Identify medication regimen, Exploit long‐term and broader health goals and Facilitate transition to independence*. Theme descriptions, accompanied by illustrative quotes selected by the researchers from the interview data, follow the thematic map. These describe the needs of young adults in relation to social robot counselling, viewed through the theoretical framework of Peplau's theoretical model.

### 4.1 The Orientation Phase: Building Human‐Robot Relation

One challenge that participants describe is the uncertainty about how to begin the actual communication with the robot. *It's good that it starts by saying ‘Hi, how can I help you?’ Otherwise, you might wonder how to begin and who takes the first step? P5*. Without a clear and friendly introduction, it can be difficult for the participant to know who is taking the first step in the conversation. This can create a sense of uncertainty and confusion about whether it is the young adult or the robot who should initiate. In the orientation phase, young adults expressed the desire for robots to provide reliable communication and help them become familiar with their medication needs.

In the initial orientation phase, when young adults interact with robots, it is crucial that they not only provide information about dosage, side effects, administration, and so forth, but also provide constructive learning opportunities via a clear communication style, natural appearance, and so forth. This helps young adults develop skills that enable them to act independently and feel confident in the relation. This phase plays a central role in building trust, as young adults rely on the robot to provide accurate, personalised advice, especially when it comes to questions about their medication.

In conversations with young adults who are just learning to manage their medication, for example, they expressed a need for the robot to offer clear communication and a mimic that could help them feel confident about what was expected of them. *First, it was quite difficult to interpret these facial expressions. P3* In these contexts, it was also expressed that the robot did not show empathy or the ability to convey emotions. Young adults said they don't want the robot to feel too mechanical or distant. *This was a very personal reaction I had, but I thought it had such red cheeks! I was a bit like, is it somehow… embarrassed in its job? P2* To create a more accessible experience, it is important that the robot not only answers questions but also takes responsibility for the young adults' needs of empathic understanding. *I would've liked to see it use some kind of greeting or something, just to make it feel more human. P1* This was expressed among the participants when the robot was dealing with sensitive or private topics, such as questions about medication or health status.

When the robot act as a guide and helps participants, it sets a foundation for future interactions. *I do feel more relaxed with the robot, mainly because you don't have to feel judged. I could just say ‘this is how it is’ and still be able to buy it. P6* It became clear that young adults are eager for an element of human interaction that can build trust and understanding during the situation of medication counselling. However, they mentioned that during the interaction with the robot, something was missing. *It wasn't like something you'd read on a blog on the internet—it still felt like you were getting reliable information. But maybe it lacked a bit… P1* On the other side, they expressed positive outcomes related to the robot offering constructive learning opportunities that allow them to gain a better understanding of their medication, dosage, and timing…*.I got plenty of information, and I felt safe asking questions and discussing things if there was anything. Just like they usually do at the pharmacy… P3* Additionally, young adults emphasised the importance of having the robot enable them to develop essential skills for managing their medications independently.

### 4.2 The Working Phase: Utilising Human Resources

In the working phase between the young adult and the robot, it is not only about addressing immediate needs, but also about actively utilising individual resources to address long‐term and broader health goals. Young adults expect the robot to be a tool that can help them not only get information about their medication but also provide advice and guidance on related health issues. The goal in this phase is to use the robot's capabilities in a more proactive way to not only address current issues, such as giving information about side effects, but also to prepare for the young adult's autonomy and future goals that go beyond their current medication. This need was not fully met during the working phase between the young adult and the robot. *Maybe it would be good if there was some additional information saying that you can ask someone, because the ending felt a bit abrupt. It was kind of like, ‘Yeah, ask someone if you have questions, bye. P1* When the robot provides information that feels reliable and useful, this strengthens the participant's trust in the technology. *When it provided the information itself, you really felt that you could trust it and you knew it was reliable information*. *P2* This suggests that young adults seek reassurance that the robot is providing accurate and relevant information, which makes them feel that they can use it as a reliable tool to address their needs in the current situation.

At the same time, participants express that there are areas where the robot could help provide more detailed information, especially around more complex aspects of medication, such as side effects. The robot not only needs to provide general information, but also the opportunity for the participant to ask more specific questions about their health problems, which would make the relationship more dynamic and tailored to the participant's individual needs. In this phase, young adults want the robot to not only focus participants on current problems but also provide guidance to prepare them for future health goals, with a chance to ask questions. *I didn't ask about the side effects, and when they were just listed like that, it could feel quite overwhelming. So maybe that's where you should be able to ask more about the side effects. P5* The robot should therefore act as a guide to managing future health issues, which are not necessarily directly related to medication, but which are still important to the individual's overall health and well‐being.

The working phase is a time when young adults want to be able to use the robot as an active and comprehensive tool to not only manage their needs in the current situation, but also to be supported in planning and preparing for future health issues*. I can imagine being served by a robot, as long as I get answers to my questions. I think it really comes down to getting the information I need without having to leave the pharmacy feeling uncertain*. P5 To achieve this, the robot has to offer both reliable and sufficient information, as well as allow the participant to explore different aspects of their health, allowing it to support the individual's long‐term goals and wellbeing.

### 4.3 The Termination Phase: Ending Human‐Robot Relation

In the termination phase of the relation between young adults and the robot, the goal is for participants to feel independent and confident in managing their medication, without being dependent on the robot. This means that through their previous interactions with the robot, they have developed the knowledge and skills needed to autonomously take control of their health and medical routines. *It's also about the ending. It did say some kind of closing phrase, like thank you or something… it was like, ‘Ah, should I take this and go?’… but it did do that, and I wouldn't say I felt uncertain…* P3 This indicates that although the closure was clear, it was something that participants were aware of, which reflects a certain degree of independence in the relation with the robot. …*And if your period doesn't start, should you consider that you might be pregnant? Things like that you can leave for people to think about… but it was good that it mentioned you can contact your contraceptive counselling, and it's really great to know where to turn*. *P5* This is how the robot can act as a guide to dealing with future health issues, also in situations related to medication, but are still important to the young adult's overall health and well‐being.

When the robot fulfils its role, and the young adult feels ready to manage their medication on their own, the relationship between human and robot can be terminated. For young adults, this marks a step forward, as they become more confident and capable of taking control of their health needs. However, participants expressed needs of a closure before the counselling could end. *It might be good to have something in writing. When I go home now, all I have with me is the package leaflet, and that can be quite scary*. P5 Although the robot plays an important role in helping young adults become more independent, participants point out that certain aspects of the conclusion could be improved to create a more complete and satisfying relation. *At the end it didn't ask me if I had any questions. So it ended quite abruptly there… it could have asked if there was anything else I was wondering about. P5* This suggests that a confirmation or the opportunity to ask more questions would have made the termination more cohesive and the relationship in a way that feels more complete and personal. *I wouldn't say I felt uncertain. But a clear beginning and ending. It needs to be clear*. *P5*

Additionally, for the young adults to feel that the robot is no longer necessary for assistance or guidance in managing their medication, the counselling process should be embedded in a broader care context. Participants expressed a need for human presence or at least a clear signal that there is a human pharmacist to turn to if more questions arise: *Or if there's someone who even greets you afterwards or stands nearby making eye contact, so you know you can ask questions. P1*.

## 5 Discussion

The findings outline the needs of young adults in relation to social robots in medication counselling using Peplau's Interpersonal Relationship Model. By following the three phases in Peplau's model (orientation, working, and termination), three main themes were found. In the following, both practical and theoretical reflections of the findings will be discussed.

### 5.1 Meeting Young Adults' Needs—Through Orientation, Working and Termination Phases

The overarching goal of Human‐Robot Interaction is to create interactions that feel as natural and intuitive as possible (Arif et al. 2017). This study suggests that young adults see social robots as tools for guiding them through a process of medication management, starting from building knowledge, moving towards engagement with health goals, and finally, experiencing autonomy. This is consistent with previous research (Vallès‐Peris et al. 2021), which stated patients’ views of robots as automated, useful tools among healthcare. The goal for Human‐Robot Interaction was clearly reflected in participants’ responses. This means that some needs of the young adults are met in some ways. For example, the robot answers questions and provides information about follow‐up care. However, several other needs, such as having continuous discussions about side effects, remain unmet within the interaction and might instead be seen as suggestions for future robot development. Previous research (Tushe 2024) found challenges among communication in healthcare between nurses and patients, such as language barriers, emotional distress, and health literacy. This study suggests that young adults perceive different types of challenges in communication compared to the more ‘typical’ challenges found in nurse‐patient communication.

The young adults also found it difficult to articulate whether they truly wanted the relationship to resemble one with a human. Nevertheless, they clearly expressed that something was missing, indicating a lack they couldn't quite define but still sensed. This was not limited to more obvious aspects, such as a clearer greeting at the beginning or a closing phrase at the end, but also included more subtle elements. This is similar to a previous study (Nyholm et al. 2021) that stated individuals experience ambivalence towards humanoid robots in healthcare. Several young adults in this study even compared the robot's behaviour to that of a human, indicating that they expected not just functional communication, but also emotional engagement. The fact that young adults compared the robot's greeting to human interaction suggests that they expected not only functional but also emotional engagement. Perhaps this can be understood as the robot lacking certain attributes (Townsend and MajidiRad 2022) that the human is accustomed to in such a situation (Dolins 2017). Related to previous research (Arpitha Shankar and Shivakumar 2024), the robot's professional and social characteristics include reliability, safety, capacity, precision, and predictability. This shows the importance of robots being developed with the ability to initiate and maintain human interactions in a way that feels natural and engaging. However, a high level of consistency may not always be beneficial, as it can make the robot appear more mechanical and less human‐like. Further studies could explore the types of emotional support young adults expect from a robot in this context, such as empathetic expressions, tone of voice, body language, or responsive listening, and how these features respond to their needs in trust and overall user experience.

According to previous research in health care (Coeckelbergh et al. 2016; Poulsen et al. 2018; Christoforakos et al. 2021), the most important thing in communication among healthcare is an efficient technique, being direct with the patient. Gestures or body language that express the robot's confidence increase trust (Townsend and MajidiRad 2022). The young adults expressed that the robot's ability to take initiative in a friendly manner was important to reduce repetitive uncertainty and build trust. For example, beginning the conversation with a friendly greeting reduces uncertainty and builds trust. The findings from this study can also be discussed in light of a similar study (Gasteiger et al. 2020) that examined the role of greeting behaviour in human‐robot interaction in delivering medications. The findings suggest that while presence (whether virtual or physical) is important, it is not just the physicality of the robot but how it engages emotionally and socially with the user that matters. Also, previous research (Rosenberg et al. 2024) shows concerns about the robot's lack of body language, which was seen as problematic as it might limit its ability to interpret nonverbal cues. This study extends these findings, as young adults emphasised the importance of the robot to communicate in a friendly and socially appropriate manner, indicating that both verbal and nonverbal communication play a key role in building rapport and trust in Human‐Robot Interaction.

While the findings from our study align with existing research (Coeckelbergh et al. 2016; Poulsen et al. 2018) on the importance of communication and trust‐building in human‐robot interaction, there are certain limitations that should be acknowledged. First, while participants expressed a desire for emotional engagement and likened the robot's behaviour to that of a human, it is important to question whether these expectations may not be universally shared or even entirely appropriate. In addition, the subthemes related to the robot identifying the young adult's medication regimen, as well as addressing their long‐term health goals, highlight important relational aspects. Research in human‐robot interactions often shows that communication is not only about exchanging information but also about building a relationship and creating a sense of social presence (Nass and Brave 2005). When young adults expressed that ‘something was missing’ or asked for a more natural beginning and ending, it reflected a need for the interaction to feel socially meaningful and safe, not just fact‐based. This might indicate that the desire for human‐like emotional interaction could lead to overestimating the robot's capabilities and potentially creating unrealistic expectations of what a robot can and should provide in a healthcare context. Further research in the field could therefore examine the differences in young adults' needs in relation to human service and robot service in this context.

Final practical reflections, when the robot fulfils its role, and the young adult experiences autonomy and feels ready to take control of their medication, the relationship between the human and the robot can be seen as concluded. However, young adults emphasised the importance of closure at this point, noting that just verbal instructions may not feel sufficient. This indicates that although the young adults understood when the medication counselling interaction had ended, many still felt that something was missing. They needed a clear closure, something to read further, or a human caring touch, until they felt confident in taking the medication. This suggests that the robot is not perceived as a mature individual in the same way as a pharmacist would be. Some researchers state that although it is important to acknowledge a future involving human‐robot interaction, it is equally crucial to recognise that machines can never fully replace the care and relational depth offered by humans (Gallagher et al. 2016; Rosenberg et al. 2024).

In other words, this study can conclude that, at present, a social robot cannot satisfy all the needs that a young adult has when purchasing emergency contraception at a pharmacy. In addition, this study shows a gap in the robot's support, and by offering additional forms of information, could enhance the young adult's confidence and autonomy. By providing such a resource, the robot's role in supporting medication management could be more effectively concluded, leaving the user with a sense of clarity and security regarding their next steps.

### 5.2 Reflecting on Implications on Human‐Robot Interaction

Peplau (1952) middle‐range theory was seen as suitable for studying human‐robot interaction, as it emphasises the development of trust, communication, and information, as well as patient autonomy, highlighting how robots can support and respect the individual's health goals and autonomy. This study proposes that Peplau's interpersonal relations model (1952) is a promising theoretical framework for studies in contexts where communication increasingly involves agents that simulate human interaction, such as social robots. In these settings, the pharmacist and client do not merely communicate through technology (as with a phone or computer), but with the technology itself. This positions the robot as a potential complement or even substitute for human interaction. Given the successful application of this model to this study's data, further exploration in future studies is suggested, especially in light of its potential to capture relational dynamics in Human‐Robot Interaction.

The findings of this study are also in line with previous research (Arpitha Shankar and Shivakumar 2024), highlighting various challenges that limit effective interaction and relations between humans and robots in healthcare. For example, these are difficulties in usability, concerns about safety and privacy, emotional discomfort, potential for deception, and a general lack of trust (Hägglund et al. 2024). Although Peplau's theory was originally developed to describe the nurse–patient relationship, this study shows that it can also be meaningfully applied to the pharmacist–customer relationship, given their shared foundation in communication, trust and professional care. The model is adaptable to various settings, including human‐robot interactions. It provides a theoretical basis for designing and evaluating interactions between patients and social robots, ensuring that these interactions are supportive and empathetic. By applying Peplau's model (1952) to the different phases of medication counselling between a young adult and a robot, it becomes easier to identify the gaps that emerge between human relational needs and what current technology is able to offer. The model also helps to explain why some participants felt that ‘something was missing’ in their interaction with the robot.

While Peplau's theory of interpersonal relations provides a valuable lens for understanding interaction and relational dynamics in healthcare contexts, there are certain limitations to its application in this study. First, the theory was originally developed to describe nurse‐patient relationships, which differ in important ways from pharmacist‐customer interactions, both in terms of expected relational depth and the structure of the encounter. Secondly, the theory is grounded in human‐to‐human interactions, sometimes supported by technology, but not in interactions where the ‘other’ is a technological agent or tool. This distinction became particularly relevant in the analysis of this study, as some young adults perceived the robot not as a relational partner but rather as a functional tool for receiving information. This suggests that new or adapted theoretical models may be needed to fully capture the dynamics of human‐robot communication and relations in pharmacy settings. In addition, this study has some other limitations. The relatively small sample size limits the generalisability of the findings. The use of self‐reported data introduces a risk of bias, as participants' perceptions and responses may be influenced by recall, social desirability, or contextual factors (Cheng and Cheng 2023).

This research contributes to the broader discourse on the intersection of technology and human caring, emphasising the importance of maintaining interpersonal relations in the digital age. Insights from this study inform the design and implementation of social robots in healthcare, showing that they meet the needs of young adults and complement medication counselling based on caring values.

## 6 Conclusion

Peplau's Interpersonal Relationship Model was shown to be a promising theoretical framework for studies in contexts where communication increasingly involves agents that simulate human interaction, such as social robots. Based on six qualitative interviews with young adults and following the three phases in Peplau's model (orientation, working, and termination), three main themes were found. In the orientation phase, the robot should offer reliable and clear communication and a neutral appearance to the young adult to create a trusting relationship. In the working phase, the robot should utilise the individuals' resources to address their medication‐related needs and begin to anticipate goals beyond the immediate problem. Finally, in the termination phase, the robot facilitates the transition to independence for the young adult to manage their own medication without relying on the robot. In light of Peplau's model, this study can conclude that, at present, a social robot cannot satisfy all the needs that a young adult has when purchasing emergency contraception at a pharmacy. However, this research contributes to the broader discourse on the intersection of technology and human caring, emphasising the importance of maintaining interpersonal relations in the digital age.

## Author Contributions

**Malin Andtfolk:** funding acquisition, conceptualisation, data curation, formal analysis, funding acquisition, investigation, methodology, validation, writing – original draft, writing – review and editing, project administration, and visualisation. **Sara Rosenberg:** formal analysis, funding acquisition, investigation, methodology, writing – original draft, writing – review and editing, conceptualisation, project administration, and data curation. **Susanne Hägglund:** Formal analysis, conceptualisation, funding acquisition, investigation, methodology, and writing – review and editing. **Mattias Wingren:** conceptualisation, investigation, writing – review and editing, funding acquisition, and software. **Andreas Lundell:** conceptualisation, funding acquisition, software, and writing – review and editing. **Linda Estman:** writing – original draft, writing – review and editing, formal analysis, investigation, methodology, supervision, and writing – original draft.

## Ethics Statement

Ethical research practices (Finnish National Board on Research Integrity TENK 2023) were followed throughout the study. Participants gave their written informed consent to take part, and their data were pseudonymized. Participation was voluntary, and all participants were informed of their right to withdraw from the study at any time without needing to provide an explanation. According to the Finnish National Board on Research Integrity (Finnish National Board on Research Integrity TENK 2019), the study design did not require an ethical review statement from a human sciences ethics committee.

## Conflicts of Interest

The authors declare no conflicts of interest.

## Data Availability

The datasets presented in this article are not readily available because they contain non‐anonymized data that may reveal participant identifiable data. Requests to access the datasets should be directed to the first author of this study.
